# Correction: Childhood infection burden, recent antibiotic exposure and vascular phenotypes in preschool children

**DOI:** 10.1371/journal.pone.0327326

**Published:** 2025-06-30

**Authors:** The PLOS One Editors

## Notice of Republication

This article [1] was republished on June 2, 2025, to address an issue identified post-publication. Please download this article again to view the correct version.
