# Supplementary material for: Childhood infection burden, recent antibiotic exposure and vascular phenotypes in preschool children
Source: PLoS One. 2023 Sep 15;18(9):e0290633. doi: 10.1371/journal.pone.0290633 (PMC10503770; doi:10.1371/journal.pone.0290633)
Supplement: S1 Table — (DOCX) [file pone.0290633.s001.docx]

S1 Table. Baseline characteristics – parent-reported febrile days.

| Characteristics | Number of parent-reported febrile days during the first year of life | | Total |  | |
| --- | --- | --- | --- | --- | --- |
|  | None | One or more |  | p-value | |
|  | n=141 | n=634 | n=775 |  | |
| Infancy (0-4 weeks of age) |  |  |  |  | |
|  |  |  |  |  | |
| Male (n, %) | 61 (43.9) | 306 (48.6) | 367 (47.7) | 0.32 | |
| Mode of delivery (n, %) |  |  |  | 0.37 | |
| Vaginal and assisted | 114 (83.8) | 518 (75.6) | 632 (84.2) |  | |
| Caesarean section | 22 (16.2) | 96 (15.6) | 118 (15.7) |  | |
| Gestational age (w) | 39.8 (1.3) | 39.9 (1.3) | 39.9 (1.3) | 0.40 | |
| Birth weight (g) | 3529 (528) | 3546 (505) | 3543 (509) | 0.72 | |
| Birth weight (z-score) | 0.01 (1.1) | 0.004 (1.0) | 0.00 (1.0) | 0.98 | |
| Maternal age at birth (yr) | 32.9 (3.5) | 32.7 (3.5) | 32.8 (3.5) | 0.62 | |
| First child in family (n, %) | 55 (39.0) | 304 (48.6) | 359 (46.8) | 0.04 | |
| Smoke exposure (n, %) | 25 (17.9) | 119 (18.8) | 144 (18.6) | 0.80 | |
| Exclusive breastfeeding duration (days) | 70.2 (82.5) | 83.8 (86.7) | 81.4 (86.1) | 0.09 | |
| European-caucasian^a^ ethnicity child (n, %) | 97 (95.1) | 486 (91.7) | 583 (92.2) | 0.24 | |
|  |  |  |  |  | |
| Childhood at pre-school visit |  |  |  |  |  |
|  |  |  |  |  | |
| Age (yr) | 5.4 (0.5) | 5.4 (0.4) | 5.4 (0.4) | 0.61 | |
| BMI (kg/m^2^) | 15.1 (1.4) | 15.2 (1.4) | 15.2 (1.4) | 0.38 | |
| Never exposed to household smoking (n, %) | 128 (92.8) | 587 (94.8) | 715 (94.5) | 0.29 | |
| GP-diagnosed allergy (n, %) | 50 (35.5) | 278 (43.8) | 328 (42.3) | 0.07 | |
|  |  |  |  |  | |
| Parental characteristics |  |  |  |  |  |
|  |  |  |  |  | |
| Grandparent with premature CVD (n, %) | 47 (33.3) | 202 (31.9) | 249 (32.1) | 0.74 | |
| Parent with premature CVD (n, %) | 5 (3.5) | 17 (2.7) | 22 (2.8) | 0.58 | |
| Mother’s BMI (kg/m^2^) | 25.3 (4.7) | 24.7 (4.0) | 24.8 (4.1) | 0.15 | |
| Father’s BMI (kg/m^2^) | 26.2 (3.1) | 25.2 (3.1) | 25.4 (3.2) | 0.003 | |
| Mothers with tertiary education (n, %) | 64 (56.1) | 403 (69.8) | 467 (67.6) | 0.004 | |
|  |  |  |  |  |  |

^a^Child European-caucasian if both parents born in European-caucasian countries (according to Center for Statistics Netherlands

<http://www.cbs.nl/nl-NL/menu/methoden/begrippen/default.htm?ConceptID=1057> )

Values are mean (SD) unless otherwise indicated
