# Supplementary material for: Childhood infection burden, recent antibiotic exposure and vascular phenotypes in preschool children
Source: PLoS One. 2023 Sep 15;18(9):e0290633. doi: 10.1371/journal.pone.0290633 (PMC10503770; doi:10.1371/journal.pone.0290633)
Supplement: S2 Table — (DOCX) [file pone.0290633.s002.docx]

S2 Table. Recent infections and carotid artery characteristics at age 5 years.

|  |  | Carotid Intima-Media Thickness (μm) | | | Carotid distensibility (mPa^-1^) | | |
| --- | --- | --- | --- | --- | --- | --- | --- |
| GP-diagnosed infections | Model | N | Linear regression coefficient (95% CI) | p-value | N | Linear regression coefficient (95% CI) | p-value |
|  | | | | | | | |
| Last 6 months (any vs none) | Unadjusted | 81/851 | -9.6 (-19.3, 0.1) | 0.05 | 68/731 | 2.5 (-3.9, 9.0) | 0.44 |
|  | Minimally adjusted* | 78/844 | -10.0 (-19.6, -0.4) | 0.04 | 64/727 | 2.8 (-3.7, 9.3) | 0.40 |
|  | Adjustedƚ | 64/694 | -9.0 (-19.8, 1.7) | 0.10 | 51/599 | 1.3 (-6.3, 8.8) | 0.74 |
|  | | | | | | | |
| Last 6 months (number) | Unadjusted | 81/851 | -5.0 (-11.4, 1.4) | 0.12 | 68/731 | 2.3 (-1.7, 6.4) | 0.26 |
|  | Minimally adjusted* | 78/844 | -5.0 (-11.4, 1.3) | 0.12 | 64/727 | 2.4 (-1.7, 6.4) | 0.25 |
|  | Adjustedƚ | 64/694 | -4.7 (-11.8, 2.5) | 0.20 | 51/599 | 1.1 (-3.8, 5.9) | 0.67 |
|  | | | | | | | |
| Last 3 months (any vs none) | Unadjusted | 42/851 | -6.0 (-19.1, 7.2) | 0.37 | 38/731 | 2.9 (-5.5, 11.4) | 0.50 |
|  | Minimally adjusted* | 41/844 | -6.6 (-19.6, 6.5) | 0.32 | 36/727 | 3.2 (-5.3, 11.7) | 0.46 |
|  | Adjustedƚ | 31/694 | -6.1 (-21.2, 9.0) | 0.43 | 26/599 | 3.5 (-6.8, 13.9) | 0.50 |
|  | | | | | | | |
| Last 3 months (number) | Unadjusted | 42/851 | -2.3 (-12.1, 7.5) | 0.65 | 38/731 | 1.9 (-4.3, 8.2) | 0.54 |
|  | Minimally adjusted* | 41/844 | -2.4 (-12.1, 7.4) | 0.63 | 36/727 | 2.0 (-4.3, 8.3) | 0.54 |
|  | Adjustedƚ | 31/694 | -0.9 (-11.6, 9.8) | 0.87 | 26/599 | 0.7 (-6.5, 7.9) | 0.84 |
|  |  |  |  |  |  |  |  |

*Minimally adjusted: age and sex.

ƚAdjusted: age, sex, pregnancy and childhood household smoking, BMI, birth weight z-score, and SES.
