# Supplementary material for: Childhood infection burden, recent antibiotic exposure and vascular phenotypes in preschool children
Source: PLoS One. 2023 Sep 15;18(9):e0290633. doi: 10.1371/journal.pone.0290633 (PMC10503770; doi:10.1371/journal.pone.0290633)
Supplement: S3 Table — (DOCX) [file pone.0290633.s003.docx]

S3 Table. Antibiotic prescriptions and blood pressure at age 5 years.

|  |  | Systolic blood pressure (mmHg) | | | Diastolic blood pressure (mmHg) | | | |
| --- | --- | --- | --- | --- | --- | --- | --- | --- |
| Antibiotic prescription | Model | N | Linear regression coefficient (95% CI) | p-value | N | Linear regression coefficient (95% CI) | p-value | |
|  | | | | | | | |  |
| Lifetime (any vs none) | Unadjusted | 554/842 | -0.5 (-1.6, 0.6) | 0.34 | 554/842 | 0.03 (-1.0, 1.1) | 0.95 | |
|  | Minimally adjusted* | 539/819 | -0.5 (-1.6, 0.6) | 0.34 | 539/819 | 0.02 (-1.0, 1.1) | 0.97 | |
|  | Adjustedƚ | 441/683 | -0.3 (-1.5, 0.9) | 0.59 | 441/683 | 0.1 (-1.0, 1.3) | 0.84 | |
|  | | | | | | | |  |
| Lifetime (number) | Unadjusted | 554/842 | -0.01 (-0.3, 0.2) | 0.92 | 554/842 | 0.06 (-0.2, 0.3) | 0.65 | |
|  | Minimally adjusted* | 539/819 | 0.001 (-0.3, 0.3) | 0.99 | 539/819 | 0.07 (-0.2, 0.3) | 0.60 | |
|  | Adjustedƚ | 441/683 | 0.07 (-0.2, 0.4) | 0.64 | 441/683 | 0.2 (-0.1, 0.5) | 0.22 | |
|  | | | | | | | |  |
| Last 12 months (any vs none) | Unadjusted | 129/842 | -1.0 (-2.4, 0.4) | 0.16 | 129/842 | -0.4 (-1.8, 0.9) | 0.53 | |
|  | Minimally adjusted* | 127/819 | -1.1 (-2.5, 0.3) | 0.13 | 127/819 | -0.4 (-1.8, 1.0) | 0.57 | |
|  | Adjustedƚ | 99/683 | -0.5 (-2.1, 1.1) | 0.57 | 99/683 | -0.3 (-1.9, 1.3) | 0.69 | |
|  | | | | | | | |  |
| Last 12 months (number) | Unadjusted | 129/842 | -0.5 (-1.4, 0.5) | 0.33 | 129/842 | -0.3 (-1.2, 0.6) | 0.57 | |
|  | Minimally adjusted* | 127/819 | -0.4 (-1.3, 0.5) | 0.41 | 127/819 | -0.2 (-1.1, 0.8) | 0.74 | |
|  | Adjustedƚ | 99/683 | -0.001 (-1.0, 1.0) | 0.99 | 99/683 | -0.07 (-1.1, 0.9) | 0.89 | |
|  | | | | | | | |  |
| Last 6 months (any vs none) | Unadjusted | 61/842 | -0.3 (-2.3, 1.6) | 0.74 | 61/842 | -1.2 (-3.1, 0.7) | 0.20 | |
|  | Minimally adjusted* | 60/819 | -0.7 (-2.7, 1.3) | 0.49 | 60/819 | -1.4 (-3.3, 0.5) | 0.16 | |
|  | Adjustedƚ | 49/683 | -0.6 (-2.8, 1.6) | 0.61 | 49/683 | -1.2 (-3.4, 0.9) | 0.27 | |
|  | | | | | | | |  |
| Last 6 months (number) | Unadjusted | 61/842 | -0.5 (-2.0, 1.0) | 0.53 | 61/842 | -1.1 (-2.6, 0.4) | 0.14 | |
|  | Minimally adjusted* | 60/819 | -0.7 (-2.3, 0.8) | 0.36 | 60/819 | -1.2 (-2.7, 0.3) | 0.12 | |
|  | Adjustedƚ | 49/683 | -0.6 (-2.3, 1.2) | 0.52 | 49/683 | -1.1 (-2.8, 0.6) | 0.21 | |
|  | | | | | | | |  |
| Last 3 months (any vs none) | Unadjusted | 32/842 | -1.4 (-4.1, 1.2) | 0.29 | 32/842 | -1.2 (-3.8, 1.3) | 0.35 | |
|  | Minimally adjusted* | 31/819 | -2.1 (-4.8, 0.6) | 0.13 | 31/819 | -1.5 (-4.1, 1.1) | 0.27 | |
|  | Adjustedƚ | 22/683 | -1.9 (-5.1, 1.3) | 0.24 | 22/683 | -1.0 (-4.2, 2.1) | 0.53 | |
|  | | | | | | | |  |
| Last 3 months (number) | Unadjusted | 32/842 | -1.1 (-3.2, 1.0) | 0.30 | 32/842 | -1.2 (-3.3, 0.8) | 0.23 | |
|  | Minimally adjusted* | 31/819 | -1.5 (-3.6, 0.6) | 0.16 | 31/819 | -1.4 (-3.4, 0.6) | 0.18 | |
|  | Adjustedƚ | 22/683 | -1.4 (-3.8, 1.0) | 0.26 | 22/683 | -1.0 (-3.4, 1.3) | 0.40 | |
|  |  |  |  |  |  |  |  | |

*Minimally adjusted: age and sex.

ƚAdjusted: age, sex, pregnancy and childhood household smoking, BMI, birth weight z-score, and SES.
