# Supplementary material for: Childhood infection burden, recent antibiotic exposure and vascular phenotypes in preschool children
Source: PLoS One. 2023 Sep 15;18(9):e0290633. doi: 10.1371/journal.pone.0290633 (PMC10503770; doi:10.1371/journal.pone.0290633)
Supplement: S1 Appendix — (DOCX) [file pone.0290633.s004.docx]

**Appendices**

S1 Appendix. International Classification of Primary Care (ICPC) codes included in general practitioner (GP) diagnosed infections.

| ICPC | English (translated from Dutch) |
| --- | --- |
| Upper Respiratory Tract Infections | |
| H71 | Acute otitis media/myringitis |
| H72 | Otitis media with effusion |
| H74 | Chronic otitis media/other ear infection |
| R71 | Whooping cough |
| R72 | Streptococcal angina/scarlet fever |
| R74 | Acute upper respiratory infection |
| R75 | Acute/chronic sinusitis |
| R76 | Acute tonsillitis/peritonsillar abscess |
| R77 | Acute laryngitis/tracheitis |
| Lower Respiratory Tract Infections | |
| R02 | Dyspnoea *If <12mo* |
| R03 | Wheeze *If <12mo* |
| R78 | Acute bronchitis/bronchiolitis |
| R81 | Pneumonia |
| R82 | Pleuritis/pleural fluid |
| R83 | Other infectious disease respiratory tract |
| R99.05 | Aspiration pneumonia |
| Gastrointestinal | |
| D10 | Vomiting |
| D11 | Diarrhoea |
| D70 | Infectious diarrhoea, dysentery |
| D72 | Viral hepatitis |
| D73 | Assumed gastrointestinal infection |
| D88 | Appendicitis |
| Urinary tract | |
| U70 | Acute pyelonephritis/pyelitis |
| U71 | Cystitis/urinary tract infection |
| X84 | Chlamydia infection female |
| Y74 | Orchitis/epididymitis |
| Skin and soft tissue | |
| B70 | Acute lymphadenitis |
| L70 | Infectious disease musculoskeletal system |
| S10.03 | Cellulitis |
| S84 | Impetigo/impetiginization |
| Other viral | |
| A71 | Measles |
| A72 | Chickenpox |
| A74 | Rubella |
| A75 | Infectious mononucleosis |
| A76 | Viral exanthema other |
| A77 | Other viral disease |
| D71 | Mumps |
| N07 | Febrile convulsion/seizure |
| R80 | Influenza |
| S70 | Herpes zoster (shingles) |
| S71 | Herpes simplex [ex. F85,X90,Y72] |
| Sepsis-like | |
| N71 | Meningitis/encephalitis |
| Other | |
| A03 | Fever |
| A70 | Tuberculosis |
| A73 | Malaria |
| A78 | Other infectious disease |
| D22 | Worms/parasites |
| K70 | Other infection cardiovascular system |
| K71 | Acute rheumatism |
| N70 | Polio |
| N72 | Tetanus |
| N73 | Other infectious disease |
