# Supplementary material for: Childhood infection burden, recent antibiotic exposure and vascular phenotypes in preschool children
Source: PLoS One. 2023 Sep 15;18(9):e0290633. doi: 10.1371/journal.pone.0290633 (PMC10503770; doi:10.1371/journal.pone.0290633)
Supplement: Attachment — Submitted filename: Response to Reviewers.docx [file pone.0290633.s005.docx]

Response to Reviewers

PONE-D-22-01018

Childhood infection burden, recent antibiotic exposure, and vascular phenotypes in preschool children

Dear Editors,

We thank the reviewers for their generous and helpful comments on our manuscript. We have addressed each point raised by the academic editor and reviewers below and feel that the manuscript is improved. We hope that the manuscript is now suitable for publication in PLOS ONE.

Sincerely,

**Journal Requirements:**

*1. Please ensure that your manuscript meets PLOS ONE's style requirements, including those for file naming.*

*2. We note that the grant information you provided in the ‘Funding Information’ and ‘Financial Disclosure’ sections do not match.*

*When you resubmit, please ensure that you provide the correct grant numbers for the awards you received for your study in the ‘Funding Information’ section.*

**We have verified that the grant numbers for awards received for our study match and are correct.**

*3. Thank you for stating the following in the Acknowledgments Section of your manuscript:*

*"The authors would like to thank all the participating families and children, and the WHISTLER study group."
We note that you have provided funding information that is not currently declared in your Funding Statement. However, funding information should not appear in the Acknowledgments section or other areas of your manuscript. We will only publish funding information present in the Funding Statement section of the online submission form.*

*Please remove any funding-related text from the manuscript and let us know how you would like to update your Funding Statement. Currently, your Funding Statement reads as follows:*

*"The WHISTLER birth cohort was supported with a grant from the Netherlands Organization for Health Research and Development (grant nr 2001-1-1322) and by an unrestricted grant from Glaxo Smith Kline Netherlands. WHISTLER-Cardio was supported with an unrestricted strategic grant from the University Medical Center Utrecht (UMCU), The Netherlands. DB is supported by an Investigator Grant (Leadership level 1; GTN1175744) from the National Health and Medical Research Council (Australia). Research at the Murdoch Children's Research Institute is supported by the Victorian Government's Operational Infrastructure Program. The funders had no role in study design, data collection and analysis, decision to publish, or preparation of the manuscript."*

*Please include your amended statements within your cover letter; we will change the online submission form on your behalf.*

**We have amended the Acknowledgements Section to remove any potential funding information, it now reads “*The authors would like to thank all the participating families and children*”. The Funding Statement as it currently reads is correct.**

*4. In your Data Availability statement, you have not specified where the minimal data set underlying the results described in your manuscript can be found. PLOS defines a study's minimal data set as the underlying data used to reach the conclusions drawn in the manuscript and any additional data required to replicate the reported study findings in their entirety. All PLOS journals require that the minimal data set be made fully available. For more information about our data policy, please see* [*http://journals.plos.org/plosone/s/data-availability*](http://journals.plos.org/plosone/s/data-availability)*.*

*Upon re-submitting your revised manuscript, please upload your study’s minimal underlying data set as either Supporting Information files or to a stable, public repository and include the relevant URLs, DOIs, or accession numbers within your revised cover letter. For a list of acceptable repositories, please see* [*http://journals.plos.org/plosone/s/data-availability#loc-recommended-repositories*](http://journals.plos.org/plosone/s/data-availability#loc-recommended-repositories)*. Any potentially identifying patient information must be fully anonymized.*

*Important: If there are ethical or legal restrictions to sharing your data publicly, please explain these restrictions in detail. Please see our guidelines for more information on what we consider unacceptable restrictions to publicly sharing data:* [*http://journals.plos.org/plosone/s/data-availability#loc-unacceptable-data-access-restrictions*](http://journals.plos.org/plosone/s/data-availability#loc-unacceptable-data-access-restrictions)*. Note that it is not acceptable for the authors to be the sole named individuals responsible for ensuring data access.*

*We will update your Data Availability statement to reflect the information you provide in your cover letter.*

**Data are available from the Julius Center for Health Sciences and Primary Care, University Medical Center Utrecht (contact via whistler@umcutrecht.nl) for researchers who meet the criteria for access to confidential data. All data are available for external parties with appropriate agreement on use and reference to the original investigators.**

*5. Please review your reference list to ensure that it is complete and correct. If you have cited papers that have been retracted, please include the rationale for doing so in the manuscript text, or remove these references and replace them with relevant current references. Any changes to the reference list should be mentioned in the rebuttal letter that accompanies your revised manuscript. If you need to cite a retracted article, indicate the article’s retracted status in the References list and also include a citation and full reference for the retraction notice.*

**Reviewer #1:**

*Minor comment:
Why would the first child in the family have more GP-diagnosed infections (slightly counter-intuitive unless this reflects anxious parents with their firstborn).*

**We believe this does reflect the difference in first-time parents’ health literacy and health-seeking behaviours which we acknowledged in the discussion as likely confounders that are difficult to measure.**

**Reviewer #2:**

*The important confounding factor that was not discussed enough by the authors is the allergy burden in the study population. The authors found that children with GP-diagnosed allergies had more infections (both GP-diagnosed infections and parent-reported febrile days). It was not mentioned though if they had more antibiotics prescriptions or not. I suggest adding a table to compare baseline characteristics between children classified according to antibiotic use. The importance of this table originated from the fact that the main positive study findings are more about recent antibiotic use rather than GP-diagnosed infections or family-reported infections.*

**We appreciate the reviewer’s response to the manuscript. We have added the additional table of baseline characteristics of the participating children and their parents according to antibiotic prescription (Table 2, page 9, line 225).**

**Table 2. Baseline characteristics – lifetime antibiotic prescription(s)**

| Characteristics | Lifetime antibiotic prescription(s) | | Total |  |
| --- | --- | --- | --- | --- |
|  | None | One or more |  | p-value |
|  | n=345 | n=583 | n=928 |  |
| Infancy (0-4 weeks of age) |  |  |  |  |
| Male (n, %) | 147 (43.0) | 301 (52.0) | 448 (48.6) | 0.009 |
| Vaginal and assisted mode of delivery (n, %) | 291 (86.9) | 468 (83.4) | 759 (84.7) | 0.16 |
| Gestational age (w) | 40.0 (1.3) | 39.9 (1.3) | 39.9 (1.3) | 0.51 |
| Birth weight (g) | 3528 (499) | 3560 (498) | 3548 (498) | 0.36 |
| Birth weight (z-score) | -0.08 (1.0) | 0.02 (1.0) | -0.02 (1.0) | 0.16 |
| Maternal age at birth (yr) | 32.5 (5.3) | 32.4 (5.1) | 32.4 (5.2) | 0.63 |
| First child in family (n, %) | 143 (43.1) | 255 (46.4) | 398 (45.2) | 0.33 |
| Smoke exposure in pregnancy (n, %) | 57 (17.1) | 102 (18.3) | 159 (17.9) | 0.65 |
| Exclusive breastfeeding duration (days) | 88.4 (87.3) | 71.3 (80.8) | 77.7 (83.7) | 0.003 |
| European-caucasian^a^ ethnicity child (n, %) | 260 (93.9) | 398 (90.7) | 658 (91.9) | 0.13 |
| Childhood at pre-school visit |  |  |  |  |
| Age (yr) | 5.4 (0.6) | 5.5 (0.3) | 5.4 (0.4) | 0.04 |
| BMI (kg/m^2^) | 15.1 (1.3) | 15.2 (1.4) | 15.2 (1.4) | 0.26 |
| Household smoke exposure in childhood (yes) (n, %) | 18 (5.3) | 32 (5.7) | 50 (5.5) | 0.81 |
| GP-diagnosed allergy (n, %) | 90 (26.1) | 279 (47.9) | 369 (39.8) | <0.001 |
| Parental characteristics |  |  |  |  |
| Parent allergy (n, %) | 163 (55.1) | 300 (62.3) | 463 (59.6) | 0.04 |
| Grandparent with premature CVD (n, %) | 115 (33.3) | 180 (30.9) | 295 (31.8) | 0.44 |
| Parent with premature CVD (n, %) | 10 (2.9) | 16 (2.7) | 26 (2.8) | 0.89 |
| Mother’s BMI (kg/m^2^) | 24.9 (4.1) | 24.8 (4.1) | 24.8 (4.1) | 0.71 |
| Father’s BMI (kg/m^2^) | 25.3 (3.2) | 25.3 (3.0) | 25.3 (3.1) | 0.87 |
| Mothers with tertiary education (n, %) | 203 (68.8) | 323 (66.1) | 526 (67.1) | 0.43 |

^a^Child European-caucasian if both parents born in European-caucasian countries (according to Center for Statistics Netherlands

<http://www.cbs.nl/nl-NL/menu/methoden/begrippen/default.htm?ConceptID=1057> )

Values are mean (SD) unless otherwise indicated

*The association between allergy especially allergic asthma and carotid intima-media thickness in children and adolescents has been suggested by different studies. This includes the study published in 2020 by some of the authors of this study and on the same (WHISTLER) cohort. They found that children with parental allergy with or without a GP-diagnosed allergy had a significantly higher CIMT compared to those not having such a history and diagnosis. While children with only a GP-diagnosed allergy had no difference in CIMT. [1] I think it is important for the authors to mention the data about parents’ allergies in this study as well (if possible) and include both parents' and children’s allergic history in the list of potential confounders before drawing the conclusion.*

**Thank you for your comments. We agree that the association between allergy and adverse vascular characteristics has previously been shown in children, including in the WHISTLER cohort. We have added parent allergy to both baseline characteristics tables (Table 1 and Table 2). Parent allergy is associated with increasing childhood antibiotic prescription and general practitioner diagnosed childhood infections, however the mechanisms underlying this relationship remain unclear and further investigations is outside of the scope of this study. We do not include allergy (childhood GP-diagnosed or parent allergy) as an *a priori* confounder as it is unlikely to directly affect the number of antibiotic prescriptions nor childhood infections. It is possible that allergies resulting in wheeze may be inappropriately treated with antibiotics, however this is likely uncommon in The Netherlands where there is parsimonious use of antibiotics and most GPs would interpret infection-associated wheeze as of viral aetiology and therefore would not prescribe antibiotics[29]. In addition, we believe that allergic wheeze lies on the causal pathway of antibiotic treatment to increased carotid intima-media thickness (CIMT). Allergic wheeze may lead to more inappropriate prescription of antibiotics, which in turn leads to increased CIMT. In this case, adjusting for allergies (including wheeze) in the analysis would filter out the effect of antibiotics on CIMT and would be methodologically incorrect.**

**Reviewer #3:**

*The second sentence about the background of the abstract was a bit confusing to me. Please edit it.*
**The second sentence about the background of the abstract was amended for clarity, it now reads:**

**“*Severe childhood infection has a dose-dependent association with adult cardiovascular events and with adverse cardiometabolic phenotypes. The relationship between cardiovascular outcomes and less severe childhood infections is unclear*.” (page 2, line 31)**

*The introduction section:
1. Needs re-writing
2. Please present the facts with data from longitudinal studies, clinical trials, and systematic reviews.
3. Can you explain the long-term effects of childhood cardiovascular outcomes to rationalize your study better? Do these effects wane with age, and what does the existing literature state in this context?* **Thank you for your suggestions to improve the Introduction section. We have added the following clarification around the long-term effects off childhood cardiovascular outcomes in the Introduction:**

**“*Cardiovascular risk factors in childhood predict adult CVD events in a dose-response manner.[8] Preclinical vascular phenotypes, including carotid intima-media thickness (CIMT) and carotid artery distensibility, are associated with traditional CV risk factors in both children and adults, and is a strong predictor of future CV events in adults.[9–12]*” (Page 3, line 70-73)**

**We further discuss the implications of childhood cardiovascular outcomes in the Discussion section (see Page 15, line 331-334).**

*Are there any prevalence estimates on childhood cardiovascular indices that result in cardiovascular diseases in adulthood?* **There are no longitudinal data that track these CV indices (by which we assume the reviewer means the carotid IMT and carotid distensibility) from preschool and primary school age children into CVD events in adulthood; cohorts have not been in existence long enough. Such studies are underway, but the participants, who were enrolled in pregnancy, are now only 10 years old. These key data will only be available to the next generation of researchers. We have shown that hospitalisation with infection in childhood is associated, in a dose-response manner, with CVD events in adulthood (PMID: 25938548), but these were population-level data and we do not have CIMT and other individual level indices.**

*In the exclusion criteria, please explain the reasons for not including children with neonatal respiratory diseases.* **The WHISTLER study was initially conceptualized to investigate paediatric wheezing illness, and neonatal respiratory disease is a significant confounder in paediatric wheezing illnesses. This current study aimed to investigate non-severe, common illnesses in otherwise healthy pre-school children, therefore neonatal respiratory disease would preclude them from this population.**

*Sentence no 104: Please include the children's data collected from the GP record here.*

**We are not entirely clear what the reviewer is requesting. In terms of infection burden, we recorded the number of GP-diagnosed infections up to age 5 years using linked GP International Classification of Primary Care (ICPC) diagnostic codes. These data were collected and analysed after the Childhood visits at age 5 years occurred. The full method of GP data collection is stated in the section Infection Burden (Page 6, line 151-167).**
