# Supplementary material for: Childhood infection burden, recent antibiotic exposure and vascular phenotypes in preschool children
Source: PLoS One. 2023 Sep 15;18(9):e0290633. doi: 10.1371/journal.pone.0290633 (PMC10503770; doi:10.1371/journal.pone.0290633)
Supplement: Attachment — Submitted filename: PLONE-D-22-01018R1_response to reviewers.docx [file pone.0290633.s006.docx]

11 August 2023

Dear Editors

**Re: Minor Revisions to PONE-D-22-01018R1 Childhood infection burden, recent antibiotic exposure and vascular phenotypes in preschool children**

Many thanks for the opportunity to submit a revised version of pour manuscript. I apologise for the delay in resubmitting; I have been on clinical service.

**Reviewer #4:**

Suggestion/s to the author:
The author should avoid repeating the numbers that are in tables (rather refer the reader to the specific table). If it is necessary, they can emphasize some of the key numbers for the reader that they think are most important. In the 1st paragraph of the results, I see that the author was showing the median and IQR, I suggest minor edits. Median and IQR are always together, therefore, no need to be continually repeating the "IQR". Maybe the author can write something like this “The median (IQR) of GP-diagnosed infections from birth to age 5 years was 3 (1-6) and 92% of children (711/773) had at least one infection recorded. The median number of parent-reported febrile episodes in the first year of life was 5 (2-10) and 82% of children (634/775) had at least one febrile episode reported. The median number of antibiotic prescriptions from birth to age 5 years was 1 (0-3) and 63% (583/928) received at least one antibiotic prescription. Of the 842, antibiotics were prescribed to 4%, 7%, and 15% of children in the 3, 6, and 12 months preceding vascular measurements, respectively. “JUST A SUGGESTION.

This implies to the second paragraph, mean and SD are always together. The author can simply say the mean (SD) of the cohort for CIMT was 388.6 um (42.3).

Response:

We are grateful to the reviewer for taking the time to make these detailed suggestions. We have edited the text as suggested, which makes the manuscript more concise and easier to read.

I have uploaded versions of the revised manuscript with tracked changes and a clean version.

We look forward to a final deposition for this manuscript.

Kind regards

**Prof David Burgner**  BSc(Hons) MBChB DTM&H MRCP MRCPCH FRACP PhD SPR FAAHMS
(Corresponding author on behlaf of all authors)

Group Leader/Principal Research Fellow, Inflammatory Origins, Murdoch Children's Research Institute
Paediatric Infectious Diseases Physician, General Medicine, The Royal Children's Hospital
Professorial Fellow, Department of Paediatrics, The University of Melbourne
Adjunct Professor, Department of Paediatrics, Monash University

**Murdoch Children's Research Institute**
The Royal Children's Hospital, 50 Flemington Road
Parkville, Victoria 3052 Australia
**T**   [+61 3 99366730](tel:+61399366730) (MCRI)
**E**   [david.burgner@mcri.edu.au](mailto:david.burgner@mcri.edu.au)
**W**  [mcri.edu.au](https://protect-au.mimecast.com/s/zW7MCp818xsAy3xYt3ZXu0?domain=aus01.safelinks.protection.outlook.com)
